# 0732. Sequential changes in the pattern of liver architecture in the acute phase of severe sepsis under the vision of videomicroscopy. Experimental study

**DOI:** 10.1186/2197-425X-2-S1-P54

**Published:** 2014-09-26

**Authors:** IHJ Koh, JC Vieirra, J Almeida-Filho, RC Tedesco, RB Souza, AMA Liberatore

**Affiliations:** Surgery, Federal University of Sao Paulo, Sao Paulo, Brazil; Surgery, Federal University Foundation of Vale do São Francisco, Petrolina, Brazil; Morfology and Genetics, Federal University of Sao Paulo, Sao Paulo, Brazil

## Introduction

Therapeutic grounded in the specific state of microcirculatory dysfunction in septic patients is still an impediment due to technological limitations, and consequently there is little knowledge about the pathophysiological microcirculatory dynamics associated with organ failure.

## Objectives

Investigate the pattern of hepatic microcirculation during the golden period of therapy in severe sepsis.

## Methods

Wistar rats underwent severe sepsis (iv. *E. coli* 2x109 CFU, DL70-80 in 26 hours3) and under general anesthesia the dynamics of microcirculatory at the liver surface was monitored by SDF1,2 at T0,T30min and T1-T6 hours and the tissue injury by histology (T0,T2h,T6h). Saline injection was used as control.

## Results

At SDF-images is possible to visualize the hexagonal architecture of hepatic lobules with radiated sinusoidal blood flow draining into the central lobular vein between the columns of hepatocytes, showing the close interaction between microcirculation and hepatocytes. In sepsis, already since 30min (T30, T1) merges areas with enlargement of hepatocytes with dilated sinusoids and narrowing of sinusoids, suggesting broad cellular edema and sinusoids that make up those narrowed blood flow limitation. From 3 hours, there was a predominance of narrowed sinusoids with or without visible flow, associated with the appearance of coalescence of columns of hepatocytes. Up to six hours, lobular architecture became misshapen and disseminated in a crescent manner. At six hours of sepsis the lobular architecture was not anymore identifiable in the entire length. The blurring of the edges of the columns of hepatocytes by its enlargement with progression of sepsis, associated with narrowing of the adjacent sinusoids, suggested that a severe vascular and tissue dysfunction were in progress. Confronting these findings with histology, the enlarged perivascular areas were composed mostly of cytoplasm edema at the early sepsis phase and of varying stages of the cell necrosis process at the further periods. These processes occurred throughout the liver showing that the surface findings can be extensive to deeper areas.

**Figure 1:**
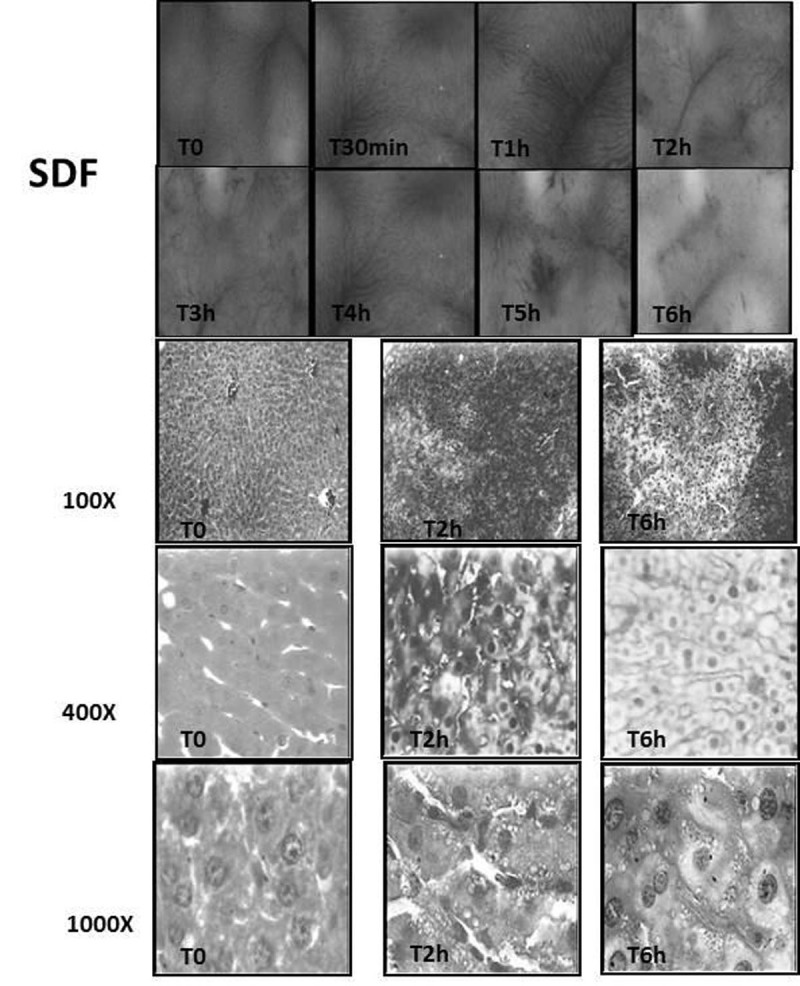
Liver images by SDF and Histology.

These findings suggested that the process involved in the genesis of hepatic failure in sepsis could be due to the cyclical repetition of the event: sinusoidal microcirculatory dysfunction - cytopathic hypoxia of the hepatocytes - edema of the columns of hepatocytes - compression of sinusoids - hepatic lobules dysfunction.

## Conclusions

The genesis of liver failure in severe sepsis appears to depend on the repetitive cyclic of sinusoidal dysfunction and subsequent adjacent hepatocytes that in turn exacerbates the progression of the liver dysfunction, thus suggesting the conjoined participation of both factors in the genesis of the solid organ dysfunction.
